# Application of benchmark analysis for mixed contaminant exposures: Mutual adjustment of perfluoroalkylate substances associated with immunotoxicity

**DOI:** 10.1371/journal.pone.0205388

**Published:** 2018-10-19

**Authors:** Esben Budtz-Jørgensen, Philippe Grandjean

**Affiliations:** 1 Department of Biostatistics, Institute of Public Health, University of Copenhagen, Copenhagen, Denmark; 2 Department of Environmental Medicine, University of Southern Denmark, Odense, Denmark; 3 Department of Environmental Health, Harvard T.H. Chan School Public Health, Boston, Massachusetts, United States of America; Utah State University, UNITED STATES

## Abstract

**Background:**

Developmental exposure to perfluorinated alkylate substances (PFASs) is associated with deficient IgG antibody responses to childhood vaccines. As this immunotoxicity outcome may represent a critical effect, calculation of benchmark dose (BMD) results would be useful for defining protective limits of exposure. However, exposures to the major PFASs that are associated with this adverse effect are interrelated, and mutually adjusted BMD results would be desirable.

**Methods:**

We carried out BMD calculations on prospective data from two prospective birth cohort studies from the Faroe Islands with a total of 1,146 children. Exposure data included serum concentrations of five major PFASs at birth and at age 5 years and, as outcome parameters, the serum concentrations of specific IgG antibodies against tetanus and diphtheria at ages 5 and 7. We calculated the BMDs and their lower confidence bounds (BMDLs) and included mutual adjustment for five major PFASs. BMD and BMDL were expressed in terms of the serum concentration of the PFASs.

**Results:**

The BMDLs for the immunotoxicants were of similar magnitude before and after adjustment. As compared to linear dose-response models, the PFASs showed lower results for a piecewise linear model, which also provided a slightly better fit. Weaker associations with the antibody outcomes were observed after adjustments due to the correlation between the PFASs. However, while the adjustments resulted in elevated BMD results and *p* values, the BMDL values were not materially changed.

**Conclusions:**

Adjustment for co-exposure to a related immunotoxicant increased both the BMD values and their standard errors, though affected the BMDL values only to a negligible extent. Thus, when correlated toxicants appear to affect the same outcome and none of them is known *a priori* to be solely responsible, all exposures may be considered responsible in BMD calculations. Our BMDL results, both before and after adjustment are generally below current exposure levels and therefore suggest that all five perfluorinated substances should attract regulatory attention, at least until additional evidence shows otherwise.

## Introduction

Perfluorinated alkylate substances (PFASs) have been in use for over 60 years in a wide array of applications [1], but it was not until the beginning of this millennium that academic research began to focus on their environmental fate, human exposures, and possible adverse effects [2]. Of note, an internal industry-commissioned study from 1978 revealed immunotoxicity in monkeys exposed to perfluorooctane sulfonate (PFOS) [3]. These findings were released to the U.S. Environmental Protection Agency many years later, and by now PFAS immunotoxicity has been demonstrated in rodent models, avian models, reptilian models, and mammalian and non-mammalian wildlife [4]. For example, reduced survival after influenza infection has been reported as an apparent effect of PFOS exposure in mice [5]. In one model, the lowest observed effect level for males corresponded to an average serum-PFOS concentration of 92 ng/mL, although 7-fold higher in females [6]. The former is similar to the highest concentrations found in serum from humans with background exposures [7].

The experimental findings triggered epidemiological studies to assess sensitive markers of immune functions. As recommended by an international group of immunotoxicity scientists, concentrations of specific antibodies against childhood immunizations were deemed to be a both feasible and appropriate outcome parameter, as children receive the routine immunizations with the exact same doses and at approximately the same age [8]. This approach would also take into account developmental vulnerability, as the first routine immunizations are usually given in early infancy when the evolving adaptive immune system is sufficiently capable of responding to antigen challenges.

Population-based serum-PFAS analyses show that PFOS and PFOA are detectable in virtually all Americans [7], with children often showing higher serum concentrations than adults [9]. Three other PFASs are commonly detected, and a government agency recently reported that it considers all five to be potentially immunotoxic in humans [10]. Paired samples of maternal serum and cord serum show that PFASs are transferred through the human placenta [11, 12], and these substances also occur in human milk [13]. Thus, long-term breastfed infants may reach serum-PFAS concentrations that are several-fold higher than the mother’s [13, 14]. The focus on vaccination responses in children vaccinated in infancy therefore appears to be highly relevant to risk assessment.

We prospectively followed birth cohorts in the Faroe Islands and showed that developmental PFAS exposures constitute a major determinant for antibody concentrations directed against tetanus and diphtheria toxoids at ages 5 and 7 years, i.e., after three or four routine vaccinations [15, 16]. Our most recent findings suggest that elevated serum-PFAS concentrations in infancy are particularly associated with lower antibody concentrations at age 5 [16]. In contrast, at age 7 years, the postnatal PFAS accumulation seems to play a major role [15]. In agreement with this observation, antibody responses to vaccinations at adult age have also been reported to be lower at elevated serum-PFAS concentrations [17, 18].

When analyzing dose-dependent adverse effects, regulatory agencies often use benchmark dose [BMD] calculations to obtain standardized points of deviation for deriving safe exposure limits [19, 20]. For example, basic PFAS toxicity data obtained from animal models have been applied to calculate BMDLs which, at first, resulted in very high levels that corresponded to serum concentrations of 23 μg/mL and 35 μg/mL for PFOA and PFOS, respectively [21, 22]. Even when taking into account large uncertainty factors, a calculated safe serum concentration would greatly exceed current levels in humans [7]. Given that immunotoxicity has been observed in humans at current exposure levels [15, 17, 23], the BMDL results obtained from routine laboratory toxicity tests are clearly insufficient to serve as a basis for safety calculations, as has also been seen in regard to other pollutants [24]. Although substantial evidence on immunotoxicity in animal models is now available [4], BMD calculations have apparently not been reported.

Until recently [25], human data on PFAS toxicity have not been considered by regulatory agencies for determining exposure limits. In a recent evaluation, the National Toxicology Program [NTP] considered the epidemiological evidence for PFOA and PFOS immunotoxicity to be only moderate, given that all studies are observational and relate to mixed exposures [26]. As at least five PFASs show adverse effects on the same target, published data do not allow a judgment if only one of them is the culprit. However, our recent calculations on birth cohort data [27] show that the effects of PFOS and PFOA appear to be independent, at least in part. However, the statistical power was insufficient to allow an assessment of potential interactions between the two exposures [27]. *In vitro* data using human white blood cells support the immunotoxic potential for several PFASs although the adverse outcome pathways may differ [28]. We now consider also three other PFASs to which humans are commonly exposed, i.e., perfluorohexanesulfonic acid [PFHxS], perfluorononanoate [PFNA], and perfluorodecanoate [PFDA].

On this background, the present report extends previous BMD results for PFAS-associated immunotoxicity [29] using an extended data base from two Faroese birth cohorts, with a wide range of background exposures to five major PFASs, now including mutual adjustment for concomitant PFAS exposures. Because the PFAS exposure levels are similar to those of other populations [7], our results should be applicable beyond the Faroes. As before, the choice of dose-response models must take into account the absence both of a known curve shape and a null exposure control group. We therefore explore different options to elucidate the impact of extrapolation beyond the exposure interval observed [30].

## Methods

### Birth cohort data

Our studies rely on birth cohorts from the fishing community of the Faroe Islands, where PFAS exposures in part originate from marine food [31]. The oldest birth cohort in this study was recruited in 1997–2000 and consisted of 656 singleton births [15]. Prospective follow-up included 587 cohort members participating in one or both examinations at ages 5 and 7 years, of whom 460 participated in both, and complete serum analyses were obtained for 431. As an exposure indicator, we used the PFAS concentrations in the mother’s pregnancy serum and in the child’s serum obtained at age 5 years. The outcomes were the specific antibody concentrations against tetanus and diphtheria toxoids in serum at ages 5 and 7 years.

The younger birth cohort consisted of 490 children born during 2007–2009 [32], so far follow-up only through age 5 years. A maternal serum sample was collected shortly after childbirth to represent the child’s prenatal exposure, and serum-antibody concentrations were obtained at age 5 (before the booster) in 349 cohort members. As comparable methods were used, we were able to combine the results from the two cohorts regarding prenatal exposure and antibody results at age 5. A total of 853 children from both cohorts had complete data that allowed them to be included in these analyses.

Among the PFASs measured in mothers and their children, PFOS and PFOA showed the highest concentrations [15, 32], and all five major PFASs showed serum concentrations that were similar to levels reported from the US [7]. Antibody concentrations showed substantial variability, many children with concentrations below the clinically protective level, despite having followed the recommended vaccination schedule with the first three vaccinations before 12 months of age.

Given the strong inverse associations between measured developmental exposures and specific antibody concentrations, we now report extended benchmark calculations first for the older cohort using the serum-PFAS concentration at age 5 as predictor of the antibody outcomes at age 7 years. These calculations correspond to our previous report [29] and are now supplemented by mutual adjustments and results on other major PFASs adjusted for PFOS and PFOA.

For the first time, we now also present the benchmark results for prenatal PFAS exposure in regard to the antibody concentration outcomes at age 5 years. These results are based on both cohorts, and again, the results for PFOS and PFOA are mutually adjusted. The two sets of calculations represent prenatal and mid-childhood exposures as different windows of immune system vulnerability. As an advantage of this population, exposures to methylmercury and polychlorinated biphenyls were only weakly correlated with serum concentrations of the PFASs [15], and confounding by these other exposures could therefore be ignored.

The cohort studies were approved by the ethical review committee serving the Faroe Islands and by the Institutional Review Board at Harvard T.H. Chan School of Public Health. All mothers provided written informed consent, and assent was obtained from the children at examinations.

### Benchmark calculations

The data were analyzed as continuous variables in SAS version 9.4 (SAS Institute, Inc. Cary, NC). Although a clinical cut-off level exists for antibody concentrations that represent long-term protection, this limit is somewhat arbitrary, and transformation of the continuous data to a dichotomous variable would result in a loss of information. Benchmark calculations were therefore based on regression models with antibody concentrations as dependent continuous variables while PFAS-concentrations were included as independent variables along with potential confounders. The benchmark methodology used [30] is outlined below.

#### Benchmark dose methodology for observational data

The BMD is the dose which reduces the outcome by a certain percentage (the benchmark response, BMR] compared to the unexposed controls [20, 21]. Different BMR values have been used in the past, and lower BMR levels are known to result in decreased BMD and BMDL results, the latter affected by increased uncertainty [31]. A 10% BMR is usually applied for experimental toxicology data [20, 21], while in human studies a BMR of 5% is often chosen [20]. As a decreased antibody response to vaccinations must be regarded an important adverse effect, the lower BMR would seem appropriate. We chose a BMR of 5% as the default [25].

The BMD was estimated in a regression models with log-transformed response variable *Y*(*d*)

logY(d)=α0+f(d)+ε, (1)

where *f* is the dose-response function satisfying *f*(0) = 0. According to its definition, the BMD will satisfy *Y*(BMD)/*Y*(0) = 1 –BMR. Taking log on both sides we get the equation log(*Y*(BMD))–log(*Y*(0)) = log(1-BMR), and according to model assumption (1), the difference on the left-hand side of this equation is f(BMD), and thus the BMD is the solution to the equation

f(BMD)=log(1−BMR) (2)

The BMD is found by estimating the dose-response function and then solving the above equation. So for a linear model [*f*(*d*) = *β* × *d*], we get BMD = log(1−BMR)/*β*. In the current application, we model the exposure effect using linear or piecewise linear dose-response functions (where the slope is allowed to change at the median exposure [29]). The BMDL is defined as the lower one-sided 95%-confidence limit of the BMD [20, 33]. In the two dose-response models considered here, exact confidence limits for the BMD can be calculated based on the fact that regression coefficients follow normal distributions [33]. These limits are computationally simple and will have correct coverage probabilities even for small sample sizes, and they are therefore preferable to more complex alternatives, such as profile likelihood or bootstrap simulations.

#### Adjustment for effects of additional variables

In the current data, the antibody response depends on a set of additional variables denoted (*x*1,…,*xn*). These are included as covariates in the regression model

logY(d,x1,…,xn)=α0+α1×x1+…+αn×xn+f(d)+ε (3)

In this more complex setup, the BMD is defined as the solution to the equation *Y*(BMD,*x*1,…,*xn*)/*Y*(0,*x*1,…,*xn*) = 1−BMR. Thus, exposure at the BMD will reduce the outcome by a certain percentage given by the BMR, as compared to unexposed controls with the same covariate values (*x*1,…,*xn*) as the exposed subject. Because of the log-linear structure of the model, it is easy to see that also in this setup the BMD must satisfy *f*(*BMD*) = *log*(1 − *BMR*). An important advantage of this definition of the BMD is that the value of the BMD will not depend on the covariates. Thus, although the response level will be affected the by the covariates, the dose that leads to a specific relative loss, will be the same for all values of the covariates under the log-linear model.

#### Application to the birth cohort data

Benchmark results were obtained for PFAS-concentrations measured at age 5 years and for prenatal exposures in the two sets of analyses, with antibody concentrations at ages 7 and 5 as the respective outcome variables. Because the younger cohort does not yet have antibody concentration measures at age 7, benchmark calculations for postnatal exposures were based on the older cohort only.

**Fig 1:**
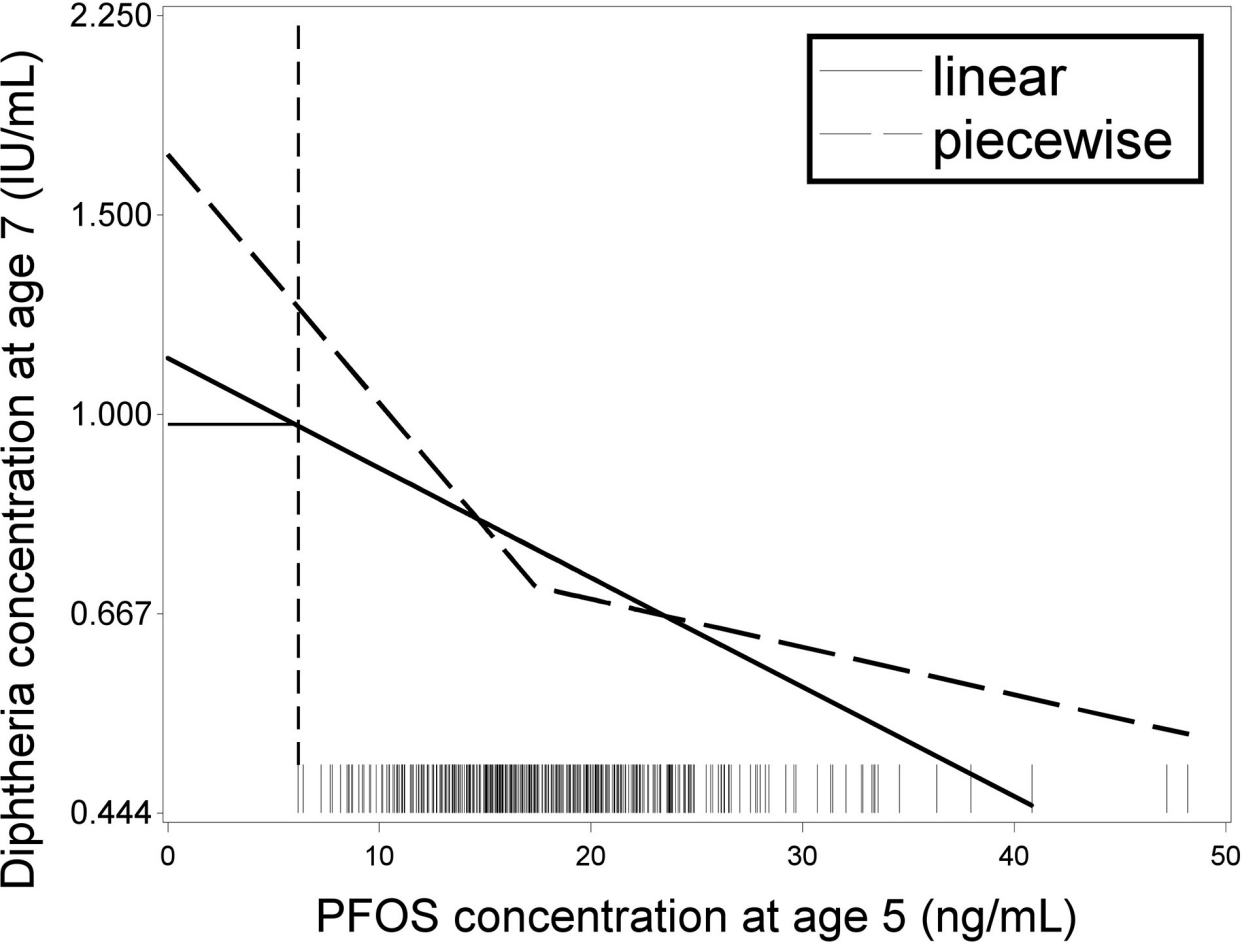
Estimated dose-response functions for the relationship between the postnatal serum-PFOS concentration and the diphtheria antibody concentration two years later.

We modeled the effects of PFAS exposure on antibody concentrations using a linear dose-response function [*f*(*d*) = *β* × *d*] and a piecewise linear model, which allowed for a difference in slopes below and above the median exposure level. The latter model is useful for benchmark calculations if the dose-response relationship at low doses differs from the one at higher doses. In such cases, a linear model would be biased, while the piecewise linear model would likely perform due to the greater flexibility. We also attempted to use a logarithmic dose-response function. However, the steep slope at very low doses may be biologically implausible, and the logarithmic curve did not show a better fit to the data compared to the piecewise linear shape. Instead, we carried out a sensitivity analysis using a conservative approach, where the slope was assumed to be flat below the lowest observed exposure (Fig 1). The benchmark dose calculation depends on the unexposed response level, and for data with no control group, an extrapolation to zero is necessary. By assuming no effect below the lowest observed concentration, the conservative model uses the most optimistic curve for extrapolation to zero exposure, and such models will therefore yield the highest plausible benchmark results that are in agreement with the data.

As the linear model is nested in the piecewise linear model, the fit of these two models was compared using likelihood ratio testing. Thus, we calculated the p-value for the hypothesis that the dose-response is linear in a test where the alternative was the piecewise linear model. Here a low p-value indicates that the linear model has a poorer fit.

The fit of the models (linear and piecewise linear) was compared by likelihood ratio testing. As the linear model is nested in the piecewise linear model, the fit of these two models was compared by calculation of the p-value for the hypothesis that the dose-response is linear in a test where the alternative is the piecewise linear model. A low p-value indicates that the linear model has a poorer fit.

As previously observed [16], relevant covariates comprised sex, age and booster type at age 5 (the latter only for the outcomes at age 7). For the analyses of prenatal exposures, regression models included observations from both cohorts, and cohort was therefore treated as a covariate. We also allowed the effect of sex and age to differ in the two cohorts by including interaction terms. Regression analysis and BMD calculations were first carried out for each of the five PFASs separately, then adjusted models were developed where the serum concentration of the relevant PFAS was included as the exposure of interest, while (log-transformed) concentrations of other relevant PFASs entered the model as additional covariate(s). Given that PFOS and PFOA are considered the PFASs with the best documentation for immunotoxicity [34, 35], the two were mutually adjusted, while the effects of PFHXS, PFNA and PFDA were adjusted for both PFOS and PFOA. Thus, in these analyses, the adjusted BMD can be interpreted as the dose level that will lead to a 5% loss in the antibody concentration when comparing an unexposed subject to one with an exposure at the BMD, where both subjects have the same level of covariates, including the PFAS(s) used for adjustment. As described in the previous section, the BMD value will be the same for all covariate profiles and will not depend on the concentrations of the PFASs used for adjustment. Thus, this model ignores possible interactions between the PFASs.

The fully drawn curve is based on the linear model, while the hatched curve is from the piecewise linear model with a break point at the median exposure level. In a sensitivity analysis, we also considered the results of a conservative model that is identical to the linear model within the range of observed exposures but assumes a horizontal curve below the lowest observed exposure level, as indicated by the dotted vertical line, i.e., that a threshold exists at the lowest serum-PFC concentration observed [29].

## Results

Both cohorts were affected by a fairly small degree of attrition, but children who participated in one clinical examination, but not the other, or not at all, did not seem to differ in terms of exposure levels from those cohort subjects who fully participated. While the duration of breast-feeding was associated with the child’s serum-PFAS concentrations [13], this parameter was unrelated to the antibody concentrations. No important confounders were identified among a wide range of social and demographic parameters, and adjustments therefore included only sex and age and, for the age-7 data, the type of booster vaccination at age 5. These covariates affected the results to a negligible degree only.

As can be seen from the (previously published [29]) left-hand columns of Table 1, the model-dependence was similar for tetanus and diphtheria antibody concentrations as outcome variables. The linear slope showed BMDL values of approximately 1 ng/mL for PFOS and PFOA, respectively, as previously reported [29]. The piecewise linear curve was steeper at low exposures, and showed BMDL results about half of those obtained by the linear dose-response curve. Mutual adjustment (right-hand columns of Table 1) resulted in higher BMD values. As the standard error also increased, the changes in the BMDL values were rather small and lesser than the differences seen between the dose-response models. All dose-response models had normally distributed residuals with a homogeneous scatter.

**Table 1:** Benchmark results for the age-5 serum concentrations of five PFASs in regard to tetanus and diphtheria antibody concentrations at age 7 years (after four vaccinations). Unadjusted benchmark results are followed by results adjusted for other serum-PFAS concentrations.

Age 7-year antibody	Postnatal exposure	Dose-response model	Not PFAS adjusted		Adjusted for other PFASs*	
			BMD	BMDL	BMD	BMDL
Tetanus	PFOS	Linear	2.70	1.31	18.99	2.03
		Piecewise	1.45	0.56	3.57	0.72
		Conservative	8.88	7.49	25.17	8.21
	PFOA	Linear	0.38	0.25	0.40	0.25
		Piecewise	0.52	0.16	0.67	0.17
		Conservative	1.70	1.57	1.73	1.57
	PFHxS	Linear	3.35	0.43	∞	0.70
		Piecewise	0.03	0.02	0.04	0.02
		Conservative	3.45	0.53	∞	0.79
	PFNA	Linear	0.33	0.15	∞	0.31
		Piecewise	0.07	0.03	0.14	0.04
		Conservative	0.72	0.54	∞	0.70
	PFDA	Linear	0.05	0.03	0.08	0.04
		Piecewise	0.03	0.01	0.03	0.01
		Conservative	0.10	0.08	0.13	0.09
Diphtheria	PFOS	Linear	2.30	1.25	3.58	1.45
		Piecewise	0.98	0.49	1.21	0.54
		Conservative	8.48	7.43	9.76	7.63
	PFOA	Linear	0.59	0.33	0.85	0.38
		Piecewise	0.48	0.17	1.06	0.20
		Conservative	1.92	1.66	2.18	1.71
	PFHxS	Linear	2.80	0.45	∞	0.64
		Piecewise	0.05	0.03	0.11	0.03
		Conservative	2.90	0.55	∞	0.74
	PFNA	Linear	0.54	0.19	∞	0.40
		Piecewise	0.06	0.03	0.14	0.04
		Conservative	0.93	0.58	∞	0.80
	PFDA	Linear	0.08	0.04	0.25	0.06
		Piecewise	0.04	0.02	0.08	0.02
		Conservative	0.14	0.09	0.30	0.11

*PFOS and PFOA are mutually adjusted, all other PFASs are adjusted for both PFOS and PFOA

Using the prenatal exposure levels and the age-5 antibody outcomes, somewhat higher results were obtained (Table 2). Because of the reliance on two cohorts, the standard error would be expected to be proportionally lower, thus resulting in a higher BMDL. The differences between the models and the effect of mutual adjustment followed the same pattern as the postnatal data (Table 1), although the differences tended to be smaller for this data set.

**Table 2:** Benchmark results for the five prenatal PFAS concentrations in regard to antibody concentrations at age 5 years (pre-booster). Unadjusted benchmark results are followed by results mutually adjusted for other serum-PFAS concentrations.

Age 5-year antibody	Prenatal exposure	Dose-response model	Not PFAS adjusted		Adjusted for other PFASs*	
			BMD	BMDL	BMD	BMDL
Tetanus	PFOS	Linear	7.25	2.86	∞	4.76
		Piecewise	2.59	1.05	∞	1.64
		Conservative	9.14	4.75	∞	6.65
	PFOA	Linear	0.55	0.32	0.59	0.31
		Piecewise	0.25	0.13	0.25	0.13
		Conservative	0.93	0.70	0.97	0.69
	PFHxS	Linear	3.11	1.37	3.90	1.50
		Piecewise	0.23	0.07	0.97	0.09
		Conservative	3.12	1.39	3.91	1.52
	PFNA	Linear	∞	0.23	∞	0.66
		Piecewise	3.55	0.14	∞	2.62
		Conservative	∞	0.38	∞	0.82
	PFDA	Linear	0.22	0.07	∞	0.09
		Piecewise	0.11	0.03	∞	0.03
		Conservative	0.25	0.10	∞	0.11
Diphtheria	PFOS	Linear	2.39	1.56	3.07	1.74
		Piecewise	1.62	0.83	2.51	0.95
		Conservative	4.27	3.45	4.96	3.62
	PFOA	Linear	0.65	0.34	2.60	0.50
		Piecewise	0.15	0.10	0.21	0.12
		Conservative	1.03	0.72	2.97	0.88
	PFHxS	Linear	1.96	1.06	2.26	1.15
		Piecewise	8.65	0.13	31.60	1.55
		Conservative	1.97	1.08	2.27	1.16
	PFNA	Linear	1.42	0.18	∞	∞
		Piecewise	0.06	0.03	0.43	0.05
		Conservative	1.58	0.34	∞	∞
	PFDA	Linear	0.09	0.05	∞	0.08
		Piecewise	0.02	0.01	0.03	0.02
		Conservative	0.12	0.08	∞	0.11

*PFOS and PFOA are mutually adjusted, all other PFASs are adjusted for both PFOS and PFOA

Table 3 shows that the various models almost equally well fit the data. Due to the greater flexibility, the piecewise linear generally tended to show slightly better fit values, but most differences observed did not reach statistical significance. However, for the postnatal effect of PFHxS on tetanus, the linear model had a significantly poorer fit and yielded benchmark results that were much higher than the piecewise linear model. In the analysis of prenatal exposures, an important difference in fit was seen for the effect of PFOA exposure on the diphtheria-specific antibody concentration. Here the advantage of piecewise linear model was significant without PFOS adjustment and borderline significant after adjustment.

**Table 3:** P-values for the hypothesis that the dose-response is linear in a test where the alternative is a piecewise linear model. Results are shown before and after adjustment for other PFASs.

		Tetanus		Diphtheria	
Sample	Exposure	Unadjusted	Adjusted	Unadjusted	Adjusted
Postnatal	PFOS	0.60	0.71	0.30	0.34
	PFOA	0.76	0.69	0.86	0.92
	PFHxS	0.002	0.05	0.05	0.44
	PFNA	0.27	0.46	0.12	0.40
	PFDA	0.51	0.40	0.55	0.73
Prenatal	PFOS	0.43	0.98	0.55	0.84
	PFOA	0.25	0.26	0.012	0.05
	PFHxS	0.45	0.90	0.70	0.11
	PFNA	0.37	0.12	0.06	0.37
	PFDA	0.81	0.84	0.05	0.12

## Discussion

Benchmark calculations are thought to provide an approximate threshold level which can be interpreted as a parallel to the No-Observed Adverse Effect level (NOAEL) from experimental studies [33]. The lower confidence limit (the BMDL) takes into account the uncertainty in the estimation of the relation between dose and effect for a given dose-response model [33]. The present report complements our previous report on BMD results for postnatal PFAS exposure [29] by adding calculations for prenatal PFAS exposures also including PFHxS, PFNA and PFDA, and with adjustments for PFOS and PFOA as the PFAS best documented as immunotoxicants. Although most regulatory agencies have so far relied on benchmark dose results obtained from animal studies of PFAS toxicity [34–36], the most recent opinion from EFSA has attempted to use BMD calculations of aggregated human data [25], although the use of deciles or quartiles can easily bias the results toward higher values. Our new calculations provide more extensive BMD results that may allow better comparison between laboratory results and epidemiological findings.

The size and homogeneity of the study population and the high participation rate are major strengths [15], as is the fact that occupational exposures and water contamination do not affect the exposure profiles in this community. PFOS and PFOA are the predominant PFASs in this population, where marine food contamination is an important source. Simple regression models for the two major PFASs may not be appropriate for mixed exposures, and our structural equation model analyses suggest that the overall effects of PFASs on the antibodies were stronger than individual effects of single PFASs [15]. The present study provides more comprehensive results.

Serum concentrations of specific IgG antibody concentrations against the two toxoid vaccines constitute well-defined immune system responses. The pre-booster concentration at age 5 represents the long-term response after the first three vaccinations during infancy, while the level two years later takes into account the response following the age-5 booster and is thought to provide long-term protection. Other clinical outcomes that reflect immune functions may be less sensitive and are also more difficult to assess in a standardized way. Still, recent studies have documented increased frequencies of common infections in small children at elevated background levels of PFASs in maternal pregnancy serum [23, 37].

While vaccine responses depend on the adaptive immune system, which undergoes important development during infancy, the most vulnerable time window for PFAS immunotoxicity is unknown. In a recent study, we modeled serum concentrations during infancy and found that levels at ages 3 and 6 months appeared to be at least as strong predictors of decreased vaccine responses at age 5 years as was the maternal concentrations [16]. The time dependence is difficult to explore in detail due to the complexity of obtaining blood samples from small children. In addition, the long elimination half-life of both PFOS and PFOA [38] means that the impact of PFAS transfer through human milk will still be apparent in serum concentrations at age 5 [13]. Thus, prenatal and early postnatal exposures will remain in the body for several years, and any age-dependent effects are difficult to separate. However, the availability of serum concentrations only at two points in time likely results in some imprecision of the exposure parameter and thereby leads to an underestimation of the associations with the outcomes [39].

An important weakness of epidemiological studies is the mixed exposures. Among the PFASs studied so far, PFOS and PFOA generally occur in serum in the largest concentrations [7], and their immunotoxic effects are well documented [4], although their adverse outcome pathways may differ [28]. In our past studies, we have utilized structural equation models to assess the total impact of the [mixed] PFAS exposures, in part to take into account the imprecision of the exposure measurements for individual PFASs [15, 27]. Joint effects of the major PFASs seemed stronger than those that could be ascribed to single compounds, and more than one PFAS therefore may contribute to the lowering of antibody responses. Given the strong experimental support for immunotoxicity of both PFOS and PFOA [4], the BMD analyses for each of the two would seem appropriate, as would mutual adjustments, as presented in the present study. However, the current regression models did not allow for a possible interaction effect between the two PFAS concentrations. If such effects are present, the calculated benchmark results may not be as protective as intended.

Given that the BMDL is calculated as the statistical 95% lower bound of the BMD, it depends on the statistical uncertainty of the BMD determination. When covariates are added to the equation, greater uncertainty will often occur, thus resulting in a greater BMD and a lower BMDL (relative to the BMD). In the current application, we adjusted the BMD results for the effect of a covariate strongly correlated with the exposure of interest. General statistical advice is to avoid inclusion of strongly correlated covariates, as the results may be over-adjusted and unstable. If inference is based on the *p* value, harmful exposures may be overlooked or disregarded as a result of inappropriate adjustment. However, these concerns may not extend to BMD analyses. Here the inference is based on the lower confidence limit, the BMDL, and over-adjustment with increased variance will likely lead to lower BMDL results that may cancel out the BMD increase. The lowered BMDL can be considered in accordance with the precautionary principle [30] and may argue against current wisdom to avoid adjustment for closely related co-exposures when BMD calculations are the purpose.

As demonstrated by our results, the choice of dose-response model results in differing BMD results, as would be expected for epidemiological studies where unexposed controls may be missing [30]. The linear curve is often used as a default, and we therefore also examined a model with a piecewise linear shape that allowed a different slope below the median exposure level. The curves fit the data about equally well. As a result, no statistical justification is available for choosing one set of results above the others. The curve shapes resulted in differences that were of similar magnitude for all five PFASs examined. These tendencies were also replicated in the results with adjustment for other PFASs.

As a consequence of the relatively steep dose-response relationships, the BMD results were mostly lower than the lowest observed exposure level. Consequently, some results depended on a part of the dose-response curve for which the data do not hold any information. The dose-response model that allowed a different slope at exposures below the median does not resolve this concern. In our previous study [29], we also included sensitivity analyses, where we assumed that no change in the antibody occurred below the lowest observed exposure level. As expected, these conservative models yielded higher BMD results, although the increase in BMDL was not substantial. Interpretation of these results must be cautious due to the questionable plausibility of the low-dose flat curve shape.

Given the differences between BMDL values for different dose-response models, different adjustments and different ages at exposure and outcomes, the present study identifies a range of BMDLs that may be useful for estimation of exposure limits. An approximate BMDL of 1 ng/mL serum for both PFOS and PFOA [somewhat higher for PFOS and lower for PFOA] would seem to appropriate. As the BMDL assumes equal sensitivity within the population studied, current guidelines [19, 20] require that the BMDL be divided by an uncertainty factor of 10 to take into account the existence of subjects with increased vulnerability. A reference concentration of about 0.1 ng/mL could then be used as the serum-based target. This concentration is below most human serum-PFAS concentrations reported [7], and it is also greatly below most previously derived BMDLs from animal toxicity tests [10]. Still, recent data on mammary gland development suggests this outcome as an additional highly sensitive target in rodents [40, 41]. When applying a standard uncertainty factor of 100, the resulting reference level is quite similar to the one that we have calculated for immunotoxicity in humans. These findings should hopefully inspire future refinement of exposure limits for PFASs.

## Conclusions

While BMD calculations on epidemiological data are normally adjusted for confounders, adjustment for correlated co-exposures is not routine. Given the need to determine exposure limits for PFOS, PFOA, and other PFASs, as likely human immunotoxicants, we calculated BMD results with adjustment for the two major PFASs. While the adjustments increased both the variance and the BMD values, they affected the BMDL values only to a negligible extent. Thus, when two toxicants appear to affect an outcome and none of them is known *a priori* to be solely responsible, the exposures should both be considered responsible. The present BMD calculations suggest that all five major PFASs should attract regulatory attention, at least until additional evidence shows otherwise.
